# Analysis of Genetic Variations in Connexin 26 ( *GJB2* ) Gene among Nonsyndromic Hearing Impairment: Familial Study

**DOI:** 10.1055/s-0042-1743257

**Published:** 2022-06-13

**Authors:** Smita Hegde, Rajat Hegde, Suyamindra S. Kulkarni, Kusal K. Das, Pramod B. Gai, Rudragouda S. Bulagouda

**Affiliations:** 1 Human Genetics Laboratory, Department of Anatomy, Shri B.M. Patil Medical College, Hospital and Research Centre, BLDE University (Deemed to be University), Vijayapura, Karnataka, India; 2 Division of Human Genetics, Karnataka Institute for DNA Research, Dharwad, Karnataka, India; 3 Laboratory of Vascular Physiology and Medicine, Department of Physiology, Shri B.M. Patil Medical College, Hospital and Research Centre, BLDE University (Deemed to be University), Vijayapura, Karnataka, India

**Keywords:** variation, familial, nonsyndromic hearing Impairment, *GJB2*, connexin 26

## Abstract

**Objective** The goal of this research was to investigate the gap junction beta 2 ( *GJB2* ) gene mutations associated with nonsyndromic hearing loss individuals in North Karnataka, India.

**Materials and Methods** For this study, patients with sensorineural genetic hearing abnormalities and a family history of deafness were included. A total of 35 patients from 20 families have been included in the study. The patient's DNA was isolated from peripheral blood samples. The *GJB2* gene coding region was analyzed through Sanger sequencing.

**Results** There is no changes in the first exon of the *GJB2* gene. Nine different variants were recorded in second exon of the targeted gene. W24X and W77X are two nonsense mutations and three polymorphisms viz. R127H, V153I, and I33T were reported along with four 3′-UTR variants. A total (9/20) of 45% of families have been identified with mutations in the targeted gene.

**Conclusion** *GJB2* mutations were identified in 19 deaf-mute patients (19/35), and 13 patients were homozygous for the mutations identified in our study cohort. In our study, W24X mutation was found to be the pathogenic with a high percentage, prompting further evaluation of the other genes, along with the study of additional genetic or external causes in the families, which is essential.

## Introduction

One in 1,000 newborns has been documented congenital hearing loss (HL), of which half are attributed to a genetic origin. In inherited deafness, the gap junction beta 2 ( *GJB2* ) gene (NG_008358) mutations are one of the single most frequent causes. 1 2 And worldwide recessive mutations in *GJB2* genes are commonly recorded in genetic HL. 3 4 Identification of genes and mutations by genetic analysis in deaf-mute children may reveal the unique behavior of several mutant alleles. Worldwide study on deaf-mute populations also recorded the *GJB2* gene involvement in causing the HL. Many of those studies involved subjects from the Indian population. 5 6 Though they included the Indian subjects, those studies failed to show the prevalence of connexin 26 mutation in the Indian cohorts. HL, though not life threatening, can become a major burden in social and professional life, and also the *GJB2* gene has emerged as the predominant cause of deafness worldwide. 3 Connexin protein contains different domains (cytoplasmic domain [CL], extracellular domains [E1–2], and transmembrane [TM1–4] domains) and all are connected in the membrane to form connexon or channels. 7 *GJB2* gene is present on chromosome 13q12, which codes for connexin 26 and it is present on DFNB1 locus. 8 9

*GJB2* is a small gene, and as such, analysis and checking for mutations is easy. For nonsyndromic congenital deaf patients, *GJB2* gene analysis gives a good starting position for mutation study. 8 Nonsyndromic hearing loss (NSHL) is a riddle that can be resolved through genetic tests, and genetic diagnosis always gives the better knowledge of abnormal and normal sensory processes. 1 To date, ∼180 genes have been identified which are associated with HL. In that, 124 genes are specifically involved in the NSHL ( https://hereditaryhearingloss.org/ ). Among these genes, *GJB2* is a major etiological cause of nonsyndromic hearing impairment. 2 A total of 444 mutations related to HL are recorded to date, in those, 355 disease-causing mutations and 51 mutations come under NSHL ( http://www.hgmd.cf.ac.uk/ac/index.php ), and they also exhibit a variety of audiometric phenotypes mild to profound. A detailed report has already been made on connexin 26 protein expression patterns correlated with the audiometric phenotypes form and in the transfected cell. 10 A mouse model study on *GJB2* gene-related deafness also showed that there are drastic changes in gap junction and decreased intercellular communication in the cochlea. 2 10 A connexin 26 protein is major protein required for normal working cochlea during the hearing. In nonsyndromic deafness (DFN), DFNB1 is the first mapped region, suffix B for the autosomal recessive inheritance. 6 *GJB2* gene mutations causing DFNB1 HL identification helps reveal a high frequency of *GJB2* mutation among NSHL patients. Since then, worldwide, most of the population demonstrated DFNB1 HL. 9 11 So, the *GJB2* gene became one of the major targets for mutation searching by the genetic diagnosis of nonsyndromic sensorineural HL. However, the mutation caused by the *GJB2* gene was majorly recorded to follow recessive inheritance, and results occurred in DFNB1 nonsyndromic hearing impairment followed homozygous or compound heterozygous pattern of inheritance. 12

## Materials and Methods

### Clinical and Audiometric Analysis

In this study, a molecular analysis of the *GJB2* gene was performed on 35 affected individuals from 20 families, who showed NSHL. All the probands were examined for HL and also for any physical illness apart from HL. Before going to the audiometric test, all the individual's information was obtained by personal interview to know clinical history, family history of HL, or any other disorders running in the family and also about consanguinity. This information was obtained after taking the informed consent from each patient.

Pure tone audiometry was done on each individual and a hearing grade was obtained based on the results. Only NSHL probands without any acquired (associated) etiology were included for the study. Probands having the sign and symptoms other than HL or any infections history such as rubella, meningitis, and history of ototoxic drugs intake during pregnancy were excluded from the study. Two to 3 mL of peripheral blood was collected in an EDTA vacutainer (BD, United States) along with the written consent and family pedigree from the patients.

### Statement of Ethics

In 2018 to 2019, ethical permission for this study was obtained by Shri B.M. Patil Medical College, Hospital and Research Centre, BLDE (Deemed to be University), Vijayapura (Ref no. BLDE(DU)/IEC/335/2018–19), and Karnataka Institute for DNA Research (KIDNAR), Dharwad (Ref no. KIDNAR/2016/07/05).

### Mutation Analysis

A DNeasy Blood and Tissue Kit was used to extract DNA from the patient's blood sample (QIAGEN, Germany). The extracted DNA's purity and amount were confirmed using a nanodrop spectrophotometer (Quawell, Q3000 UV spectrophotometer) and % gel electrophoresis, respectively. Sequencing was performed with the help of big dye terminator cycle sequencing kit V3.1 (Applied biosystem, United States) on the ABI 3500 Sanger sequencing platform. The complete coding region (exons 1 and 2) of the *GJB2* gene was sequenced. Before sequencing, the coding region was amplified with the help of a polymerase chain reaction (PCR) technique. Only purified PCR products were subjected to sequencing. Results were compared with the standard reference sequence in the NCBI database (NG_008358) to confirm the changes in the patient's nucleotide sequence. The list of primers used was tabularized in Table 1 .

**Table 1:** The list of primers used for the study

Exon	Primer ID	Nucleotide sequence
Exon 1	DM-EX1-F	CCCTCCGTAACTTTCCCAGT
	DM-EX1-R	CCAAGGACGTGTGTTGGTC
Exon 2	DM-EX2A-F	CCTGTTTTGGTGAGGTTGTG
	DM-EX2A-R	TGGGTTTTGATCTCCTCGAT
	DM-EX2B-F	CTACTTCCCCATCTCCCACA
	DM-EX2B-R	CCTCATCCCTCTCATGCTGT
	DM-EX2C-F	GTTTAACGCATTGCCCAGTT
	DM-EX2C-R	GGCACTGGTAACTTTGTCCA
	DM-EX2D-F	CCAACTTTCCCCACGTTAAA
	DM-EX2D-R	TGGCTACCACAGTCATGGAA
	DM-EX2E-F	GCACAGCTGAGAGGCTGTCT
	DM-EX2E-R	GCTGAAGGGGTAAGCAAACA
	DM-EX2F-F	GGGGAGGGAGAAGTTTCTGT
	DM-EX2F-R	AATGGGGTCAGACACTCTGG
Intron	DM-IN1A-F	CTGGACCAACACACGTCCTT
	DM-IN1A-R	GGAAACAGACCCTCGTGAAG
	DM-IN1B-F	CAGAGATTTGGGCGGAGTT
	DM-IN1B-R	TCACCAGGATCCAGAAAAGG
	DM-IN1C-F	TGCACAGTCGGTCACAATTT
	DM-IN1C-R	CCAAACCCAGGTCATACACC
	DM-IN1D-F	TCAGCTGATGGTAACTGGACA
	DM-IN1D-R	CACCAAGGTCAGGCAGAAAC
	DM-IN1E-F	TGTTGTCTTTCCCAAGCTCA
	DM-IN1E-R	TCAACTCCCTCGGTTACTGG
	DM-IN1F-F	CGCTTGCAGTAAGGAGTGTG
	DM-IN1F-R	AGGCTGAGAGGCCAAGTACA
	DM-IN1G-F	CACTGCTACATGCCACGTCT
	DM-IN1G-R	TCTTCCTGAGCAAACACCAA

Note: The coding and noncoding regions of the *GJB2* gene (complete gene) were amplified with the help of 14 sets of primers.

### Insilico Analysis for *GJB2* Gene

We used bioinformatics-based methods to predict the effect of nucleotide changes recorded. dbSNP, 1000 Genome, ExAc, and ClinVar databases were used to identify the variations which are already recorded by different studies. Pathogenicity of the recorded mutations was checked by the different insilico analysis tools such as PolyPhen-2, 13 PANTHER, PROVEAN, PhD-SNP, SNPs&GO, and SNAP2. Evolutionary conservation of Connexin 26 protein sequence analyzed using the ConSerf server.

## Results

### Clinical and Audiometric Results

The patient's history and physical examinations did not show any environmental factors influencing deafness, confirming the nonsyndromic form of deafness. All patient's hearing tones, severity, and types were revealed by the audiological outcome (200–8,000 Hz). The hearing level and degree of HL were defined, according to the mean. Up to 25 dB (hearing level measured in decibels [dB]) Normal hearing is defined as 26 to 40 dB; mild hearing is defined as 41 to 70 dB; moderate hearing is defined as 71 to 90 dB; severe hearing is defined as >90 dB; and profound hearing is defined as >90 dB. 14

### Molecular Study Results

Nine different variants (n _family_ = 20, n _pateints_ = 35) were identified in our study cohort. They included two pathogenic nonsense variations, three missense variations, and four 3′-UTR variations (shown in Table 2 ). c.71G > A nonsense pathogenic variants recorded in nine affected individuals (9/35). In nine affected individuals, one patient was heterozygous for c.71G > A and c.380G > A variants. Remaining eight were identified as homozygous for c.71G > A (p.Trp24Ter). Three missense variations viz. c.380G > A, c.457G > A, and c.98T > C were identified in five individuals. Two individuals were occurred in heterozygous state for c.457G > A (p.Val153Ile), and other two were heterozygous for c.380G > A (p.Arg127His) variant. Remaining one was homozygous. Addition to this four, different 3′-UTR variants were identified in the five affected individuals.

**Table 2:** Genetic variation recorded in our study cohort

Gene	dbSNP	Nucleotide sequence variant	Effect on protein	Protein domain	Function consequences	Clinical significant	N/R
*GJB2*	rs104894396	c.71G > A	W24X	TM1	p.Trp24Ter (nonsense mutation)	Pathogenic	R
	rs80338944	c.231G > A	W77X	TM2	p.Trp77Ter (nonsense mutation)	Pathogenic	R
	rs575453513	c.98T > C	I33T	TM1	p.Ile33Thr (missense mutation)	Possibly damaging	R
	rs111033196	c.380G > A	R127H	CL	p.Arg127His (missense mutation)	Benign	R
	rs111033186	c.457G > A	V153I	TM3	p.Va153Ile (missense mutation)	Benign	R
	rs3751385	c.*84T > C	–	–	3′-UTR variant	Benign	R
	rs9237	c.*1067G > T	–	–	3′-UTR variant	Benign	R
	rs7988691	c.*1277T > C	–	–	3′-UTR variant	Benign	R
	rs7623	c.*1152G > A	–	–	3′-UTR variant	Benign	R

Abbreviations: CL, M2-M3 cytoplasmic loop; N, new; R, recorded; TM1, transmembrane domain 1; TM2, transmembrane domain 2; TM3, transmembrane domain 3.

Note: The position of the nucleotides following the 3′ of the translation loop codon is indicated by the c* number. The genotype frequencies are based on the North Karnataka population.

### Family Pedigree Analysis

**Fig. 1:**
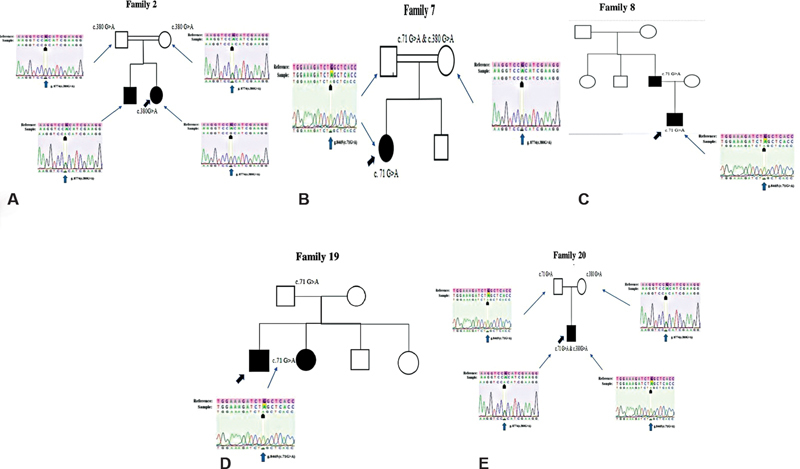
Five-family pedigree. The graphic depicts *GJB2* gene mutations ( **A–E** ). ( **A** ) Pedigree and electropherograms of the missense mutation c.380G > A in Family 2. ( **B** ) A pedigree demonstrating the inheritance pattern of the nonsense mutation c.71G > A in Family 7. ( **C** ) A pedigree demonstrating the inheritance pattern of the nonsense mutation c.71G > A in Family 8. ( **D** ) Pedigree showing the pattern of inheritance of the nonsense mutation c.71G > A (p.Trp24Ter) in Family 19. ( **E** ) The pedigree of Family 20 demonstrates the inheritance pattern of a compound heterozygote of c.71G > A and c.380G > A, as well as electropherograms. (The pedigree was created with the visual paradigm online diagram tool [ https://online.visual-paradigm.com/drive/#diagramlist:proj=0&new=PedigreeChart ]).

To explore the pattern of inheritance of coding region mutations c.71G > A, c.231G > A, and 380G > A, all members of five families (Family 2, Family 7, Family 8, Family 19, and Family 20) were submitted to Sanger sequencing, and their pedigrees are illustrated in Fig. 1 . The potentially damaging c.71G > A and c.231G > A genotypes are passed down as a homozygous recessive manner. In Family 2, the proband was homozygous for c.380G > A, and his impacted sibling was heterozygous (as indicated in Table 3 ), but their unaffected father was homozygous and their unaffected mother was heterozygous for the same variation. The mutation c.380G > A is not a pathogenic variant, thus the causative gene in this family must be distinct from the chosen gene. In Family 7, the proband was homozygous, while parents were heterozygous for c.71G > A variant. In the instance of Family 8, the proband was homozygous for c.71G > A, and his father, who also had NSHL, did not reveal any pathogenic variations in the genes included for this analysis, indicating that other genes may be involved in HL. The proband and both of his afflicted siblings were determined to be homozygous for the c.71G > A pathogenic mutation in Family 19. And his other two siblings were perfectly normal. Because of newborn screening and subsequent treatments, the proband can converse vocally. Family 20 is the perfect example of a compound heterozygous inheritance pattern. The proband is compound heterozygous for both the c.71G > A and the c.380G > A alleles. Each heterozygous parent passed on these variations to the proband. In our cohort, the most prevalent mutations in the *GJB2* gene were c.71G > A and c.380G > A. Because of the great diversity of autosomal recessive NSHL, epidemiological investigations across a diverse range of ethnic groups are required to determine the prevalence of GJB2 mutations as a cause of hearing impairment.

**Table 3:** Clinical features of probands and family details

Patient code		Sex/age	Clinical feature	Heavy medication history	HL level	HL type	Age onset	Variant finding
Family	Pedigree							
DMF2	Father	M/45	Healthy	No	Normal	BN	NK	c.380G > A
	Mother	F/30	Healthy	Yes (second pregnancy)	Normal	BN	NK	c.380G > A
	Brother	M/13	NSHL	–	Moderate	Unilateral HL (right ear)	6 y	
	Proband	F/10	NSHL	–	Profound	Bilateral sensorineural high-frequency HL	By birth	
DMF7	Father	M/35	Healthy	No	Normal	BN	–	c.71G > A
	Mother	F/30	Diabetic	Yes	Normal	BN	–	c.380G > A
	Brother	M/10	Healthy	–	Normal	BN	–	–
	Proband	F/7	NSHL	–	Profound	Bilateral sensorineural HL	By birth	c.71G > A
DMF8	Grand father	M/70	Healthy	–	Normal	BN	–	–
	Grand mother	F/60	Healthy	–	Normal	BN	–	–
	Father	M/48	NSHL	–	Severe	Bilateral HL	7 y	c.71G > A
	Mother	F/40	Healthy	No	Normal	BN	–	–
	Proband	M/15	NSHL	No	Profound	Bilateral sensorineural HL	By birth	c.71G > A
DMF19	Grand father	M/75	Healthy	–	Normal	BN	–	No DNA available
	Grand mother	F/61	Healthy	–	Normal	BN	–	No DNA available
	Father	M/40	Healthy	No	Mild	Unilateral HL(left ear)	NK	c.71G > A
	Mother	F/36	Healthy	–	Normal	BN	–	–
	Sister 1	F/18	Healthy	–	Normal	BN	–	–
	Brother	M/15	Healthy	No	Mild	Bilatreal HL	NK	No DNA available
	Sister 2	F/10	NSHL	No	Severe	Bilateral sensorineural HL	8 y	c.71G > A
	Proband	M/8	NSHL	No	Profound	Bilateral sensorineural high-frequency HL	By birth	c.71G > A
DMF20	Father	M/50	Healthy	–	Normal	BN	–	c.71G > A
	Mother	F/39	Healthy	–	Normal	BN	–	c.380G > A
	Proband	M/6	NSHL	No	Profound	Bilateral sensorineural high-frequency HL	By birth	c.71G > A & c.380G > A

Abbreviations: BN, bilateral normal; HL, hearing loss; NK, not known; NSHL, nonsyndromic hearing loss.

## Discussion

**Fig. 2:**
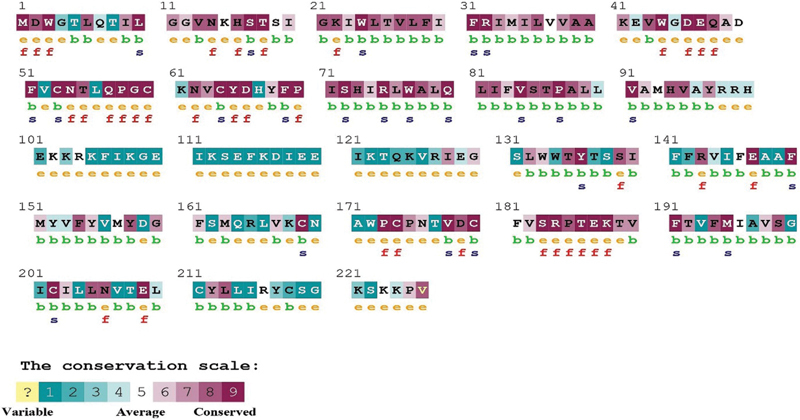
Protein (connexin 26) conservation of p.W24X and p.R127H (p.W24X is highly conserved and buried, and p.R127H mutation is variable).

We conducted this research to discover the gene variants that cause NSHL in the North Karnataka population of India. This is the first kind of screening program conducted on the NSHL population of the North Karnataka region. Heterogenicity in genetic HL and also the involvement of different alleles in HL in different populations were major reasons for conducting this study. 15 16 17 18 19 20 In our study group, pathogenic variants specific to the *GJB2* gene were identified in 9 individuals out of 35 affected individuals (28%). Various genetic research on HL have also revealed that the c.71G > A (W24X) mutation is the most common pathogenic variation causing NSHL in India. 5 19 21 In this study also, 8 (8/35) probands showed c.71G > A (W24X) mutation. This mutation is present on TM1 of the connexin 26 protein. As a result, the protein was truncated to one-tenth the sequence of the wild-type protein. This is due to the G > A transition at c.71, which results in a stop codon at p.24 (W24X) of connexin 26. The bioinformatics results also support the deleterious effect of CX26 protein by this mutation (shown in Table 4 ). This c.71G > A dominance in the Indian population might be due to the founder effect. 5 21 Other variants identified in this study were c.380G > A (R127H) and c.457G > A (V153I), which were classified as “ *others* ” because protein function prediction tools such as PROVEAN and PolyPhen-2 classified these variations as benign, whereas PhD SNP, SNPs&GO, SNAP2, and PANTHER classified them as pathogenic 22 (shown in Table 4 ). c.380G > A (R127H) mutation found on the cytoplasmic domain of CX26, which affects the residue is not highly conserved among the β connexin. 22 This implies the nonpathogenic nature of R127H mutation shown in Fig. 2 . However, functional studies of this variant were nonconsistent. 23 Previous studies conducted on HL in India also recorded c.380G > A (R127H) mutation in high frequency, 5 23 24 25 like in this study, 5 probands were recorded with c.380G > A variant (5/35). Our pedigree study confirms that the c.380G > A variation is not pathogenic and does not induce HL in the families. Previous research on the Indian population backs up this claim. 5 23 25 In this study, another mutation was recorded which is also involving a premature stop codon resulting in nonsense mutation c.231 G > A (W77X). Only a single proband has been diagnosed with this mutation (1/35), even though the frequency of this mutation was very less in previously reported HL subjects. 5 12 26 The commonly found GJB2 mutation c.35delG in white and c.235delC in Chinese and Japanese were surprisingly not seen in our cohort. 16 17 20 Although the variant c.35delG was reported in the North Indian population, 27 the frequency of the mutation was low compared with the W24X and R127H mutations. We have found four 3′-UTR variants in *GJB2* in our study group, but no functional analysis was conducted on these UTR variants.

**Table 4:** Clinical significance of identified *GJB2* variant by insilico analysis

Variant	dbSNP	PROVEAN	PhD-SNP	x	SNPs&GO	CADD	DVD
I33T	rs575453513	Deleterious Score: −3.722	Disease P: 0.548	Possibly damaging Score: 0.792	Disease P: 0.548	25.2	Pathogenic
V153I	rs111033186	Neutral Score: −0.205	Neutral P: 0.149	Benign Score: 0.003	Neutral P: 0.083	23.4	Benign
R127H	rs111033196	Neutral Score: −0.786	Disease P: 0.658	Benign Score: 0.001	Disease P: 0.589	23.2	Benign

Abbreviations: CADD, combined annotation dependent depletion.

Note: *PROVEAN* : If the prediction score is −2.5, the impact is “destroys.” *SNPs&GO* : If the probability is more than 0.5, it is expected to be a disease-causing nsSNP. *PolyPhen-2* : With a score close to 1, the most disease-causing capacity is “probably damaging.” With a score of 0.5 to 0.8, “possibly damaging” has less disease-causing capacity. “Benign” means that it has no effect on protein functions and has a score close to 0. *PHD-SNP* : If the likelihood is more than 0.5, the mutation is projected to be “disease,” and if the probability is less than 0.5, the mutation is anticipated to be “neutral.”

The frequency of W24X mutation was found to be very high in our study population, and the absence of c.35delG, c.235delC, and 167delT mutations could be population specific.

## Conclusion

The current study's findings show that mutations in the *GJB2* gene are a substantial contributor to NSHL in the North Karnataka community, which varies from the findings of other ethnic groups' studies. Nineteen (54%) deaf-mute patients were detected with the *GJB2* mutations. Out of that, 13 (37%) patients were homozygous. W24X and R127H mutations were recorded in high prevalence in our study group compared with the other missense variations and 3′-UTR variations. W24X mutation was recorded as pathogenic and R127H mutation was recorded as benign. Thus, W24X mutation in the *GJB2* gene appears to play a major role in familial deafness. Further investigation of the other discovered gene regions, as well as the search for other genetic reasons in the genetic deafness family group, is required. This study shows that investigation of the *GJB2* gene is a preliminary step before going to next-generation sequencing. 9 We also wanted to add that analysis of genes using Sanger sequencing for mutation study may be economical and also be a faster diagnostic technique.
